# Numerical Investigation of GaN HEMT Terahertz Detection Model Considering Multiple Scattering Mechanisms

**DOI:** 10.3390/nano13040632

**Published:** 2023-02-05

**Authors:** Qingzhi Meng, Qijing Lin, Zelin Wang, Yangtao Wang, Weixuan Jing, Dan Xian, Na Zhao, Kun Yao, Fuzheng Zhang, Bian Tian, Zhuangde Jiang

**Affiliations:** 1 State Key Laboratory of Mechanical Manufacturing Systems Engineering, Xi’an Jiaotong University, Xi’an 710049, China; 2 Collaborative Innovation Center of High-End State Key Manufacturing Equipment, Xi’an Jiaotong University, Xi’an 710054, China; 3 Shandong Laboratory of Yantai Advanced Materials and Green Manufacturing, Yantai 265503, China; 4 Xi’an Jiaotong University (Yantai) Research Institute for Intelligent Sensing Technology and System, Xi’an Jiaotong University, Xi’an 710049, China

**Keywords:** terahertz detector, detection model, response voltage, scattering mechanism, material property

## Abstract

GaN high-electron-mobility transistor (HEMT) terahertz (THz) detectors have been widely studied and applied in the past few decades. However, there are few reports about the influence of GaN/AlGaN heterostructure material properties on the detection model at present. In this paper, a response voltage model for a GaN HEMT THz detector that considers the carrier scattering in a GaN/AlGaN heterostructure is proposed. The phonon scattering, dislocation scattering, and interface roughness scattering mechanisms are taken into account in the classic THz response voltage model; furthermore, the influence of various material parameters on the response voltage is studied. In a low-temperature region, acoustic scattering plays an important role, and the response voltage drops with an increase in temperature. In a high temperature range, optical phonon scattering is the main scattering mechanism, and the detector operates in a non-resonant detection mode. With an increase in carrier surface density, the response voltage decreases and then increases due to piezoelectric scattering and optical phonon scattering. For dislocation and interface roughness scattering, the response voltage is inversely proportional to the dislocation density and root mean square roughness (RMS) but is positively related to lateral correlation length. Finally, a comparison between our model and the reported models shows that our proposed model is more accurate.

## 1. Introduction

Terahertz (THz) detection technology has attracted a lot of attention for its applications in the areas of noninvasive imaging [1,2,3], biomolecular detection [4,5,6], material composition detection [7,8], etc. Among various types of THz detectors, GaN high-electron-mobility transistor (HEMT) THz detectors have been widely studied [9,10,11,12,13,14] in the past few decades due to their advantages of high sensitivity, fast response time, and micro–nano size. For the design and the evaluation of detectors’ performance, it is necessary to establish an accurate THz detection model.

The first THz detection model for an HEMT detector was proposed by M. Dyakonov and M. Shur [9,10], who theoretically predicted that an HEMT could be used as a THz detector. It is demonstrated that the THz wave is coupled into the conductive channel, which inspires the plasmons to generate a rectification signal between the source and drain electrodes. This classic model details the detection mechanisms of resonant and non-resonant modes for field-effect transistor (FET) THz detectors, including GaN or GaAs-based HEMTs, revealing the relationship between the response voltage and gate length, operating temperature, or oscillation frequency. Additionally, many researchers have studied this model in depth during the past few decades, e.g., the developed THz detection model taking into account the electron transport and gate leakage current [15], the non-resonant detection model that includes dc conductivity [16], the detection model considering the circuit and parasitic effects [17], the electrodynamic model for FET THz detectors containing the influence of electromagnetic wave propagation [18], etc. However, the present models mainly focus on the aspects of external circuits and the electrical performance of an FET to improve the precision, and the influence of epitaxial-layer material properties on a THz detection model has seldom been studied. For GaN HEMT THz detectors, the quality of the GaN/AlGaN epitaxial layer determines the intrinsic properties of devices, possibly even the upper limitation of the device performance. Therefore, if the relationship between the GaN/AlGaN epitaxial-layer material parameters (e.g., dislocation density, interface roughness) and two-dimensional electron gas (2DEG) plasma characteristics can be established, it will be beneficial for the design of GaN HEMT and other similar types of THz detectors.

In this paper, we propose a response model for a GaN/AlGaN HEMT THz detector, considering the carrier scattering mechanisms. We investigated the average freedom time under the action of phonon scattering, dislocation scattering, and interface roughness scattering mechanisms. The effects of temperature, 2DEG and dislocation density, and interface roughness on the response voltage of GaN HEMT THz detector are numerically calculated. Finally, the accuracy of the proposed model is validated by comparing the theoretical calculation results with our previous experimental results and the reported models.

## 2. Model Derivation

### 2.1. Basic Equation

**Figure 1:**
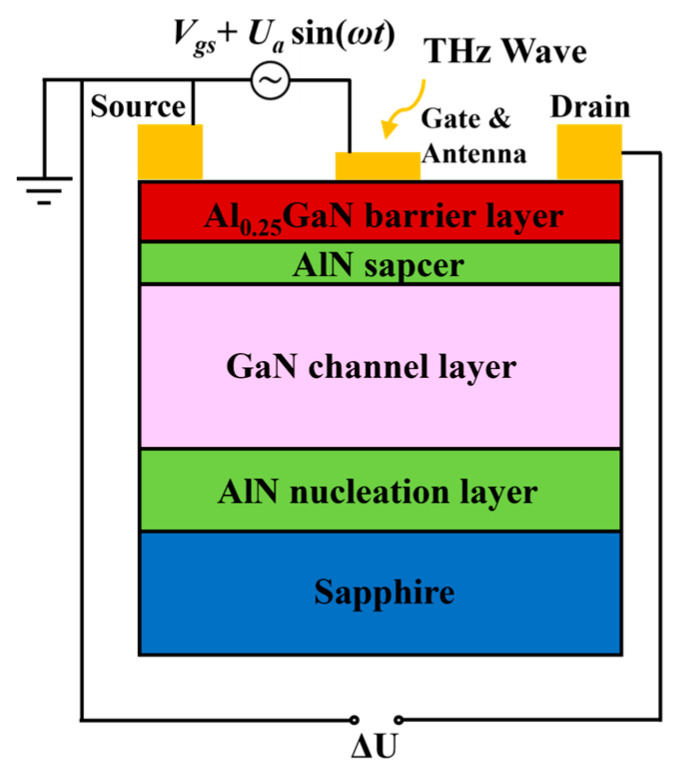
Diagram of a typical GaN HEMT THz detector and equivalent circuit diagram for THz wave detection. THz wave is collected by antenna connected to gate electrode, and response voltage Δ*U* is generated between source and drain.

According to FET THz detection theory, THz wave is collected by an antenna and coupled into the FET channel, which excites the 2DEG plasma oscillation in channel, resulting in a voltage difference Δ*U* between the source and drain electrodes. A typical GaN/AlGaN HEMT THz detector structure and its equivalent circuit diagram is shown in Figure 1. The voltage between the gate and source is composed of a DC bias *V_gs_* and an AC signal *U_a_* cos(*ωt*) induced by THz wave, and the source is grounded. *U_a_* is the amplitude related to the intensity of the THz electromagnetic field and *ω* is the angular frequency of the incident THz wave.

Assuming that GaN HEMT is a gradual channel approximation, the surface density of 2DEG *n_s_* is

(1) $$n_{s}=CU/e$$

where *C* is the capacitance per unit area between gate and channel, *U* is the local potential between the gate and channel, and *e* is electron charge.

The 2DEG motion in the channel is described by a hydrodynamic Euler equation and current continuity equation [10]

(2) $$\frac{\partial v}{\partial t}+v\frac{\partial v}{\partial x}+\frac{e}{m^{*}}\frac{\partial U}{\partial x}+\frac{v}{\tau }=0$$

(3) $$\frac{\partial n}{\partial t}-\frac{1}{q}\frac{\partial j_{n}}{\partial x}=0$$

where *v* is the velocity of local electron, *x* is the direction parallel to the channel, m* is the electron effective mass, *τ* is the average freedom time, and *j* is the current density. The boundary conditions of Equations (2) and (3) are

(4) $$U(0,t)=U_{0}+U_{a}\cos(\omega t)\;\;\;\;\;\;\;\;\;\;x=0$$

(5) j(L,t)=0                       x=L

where *U_a_*cos(*ωt*) represents the AC signal induced by the THz wave, and *U*_0_ = *U_gs_* − *U_th_* and *L* are the gate length. By solving Equations (2) and (3) through the boundary conditions, the expression of the response voltage Δ*U* is obtained as [10]

(6) $$\frac{\Delta U}{U_{0}}=\frac{1}{4}{\left(\frac{U_{a}}{U_{0}}\right)}^{2}f(\omega )$$

where *f*(*ω*) is expressed as

(7) $$f(\omega )=1+\beta -\frac{1+\beta \cos(2k_{0}^{\prime }L)}{{\sin h}^{2}(k_{0}^{\prime \prime }L)+\cos^{2}(k_{0}^{\prime }L)}$$

where *β*, $k_{0}^{\prime }$, and $k_{0}^{\prime \prime }$ are

(8) $$\beta =\frac{2\omega \tau }{\sqrt{1+{\left(\omega \tau \right)}^{2}}}$$

(9) $$k_{0}^{\prime }=\frac{\omega }{s}{\left(\frac{(1+\omega^{-2}\tau^{-2})+1}{2}\right)}^{1/2}$$

(10) $$k_{0}^{\prime \prime }=\frac{\omega }{s}{\left(\frac{(1+\omega^{-2}\tau^{-2})-1}{2}\right)}^{1/2}$$

where *s* = (*eU*_0_/*m*)^1/2^ is the plasma wave velocity. Equations (6)–(10) are a general response model for both resonant and non-resonant modes. The parameters *U_a_* and *ω* are related to the incident THz radiation, and *τ* is determined by the inherent material characteristics of the GaN/AlGaN epitaxial layer. In previous works [15,16,17,18], *τ* is set as a constant value for the calculation of the response model. However, *τ* is actually a parameter closely related to material parameters and carrier scattering in the channel. Therefore, investigating the influence of scattering mechanisms in GaN/AlGaN heterostructures on the response model is indispensable.

### 2.2. Scattering Mechanisms

#### 2.2.1. General Equation

For semiconductor materials, carrier scattering can be divided into two categories: the intrinsic scattering mechanism that all types of crystals are comprised, such as acoustic or optical phonon scattering, and the scattering mechanism resulting from the lattice mismatch during crystal growth or intentional doping, such as dislocation scattering, interface roughness scattering, or ionized impurity scattering. For GaN/AlGaN HEMT, 2DEG is formed in the triangular barrier of GaN/AlGaN heterostructure due to the piezoelectric polarization effect. In the electron phonon scattering mechanism, the deformation potential scattering, piezoelectric potential scattering, and optical phonon scattering must be considered. Furthermore, as the dislocation penetrates through the GaN/AlGaN heterostructure and the height fluctuation at the GaN/AlGaN interface, the dislocation scattering and interface roughness scattering should also be calculated. To simplify the model, we assume that the 2DEG in GaN/AlGaN HEMT is completely formed by spontaneous polarization without any intentional doping, and that the Al component is supposed to be uniformly distributed. Consequently, the ionized impurity and alloy scattering is ignored.

The general equation describing the carrier scattering rate 1/*τ_j_*, the reciprocal of the average freedom time, is given as [19,20]

(11) $$\frac{1}{\tau_{j}}=A_{j}\times {\iint_{\theta ,E}\left(\frac{M_{j}}{S\left(q,T,E\right)}\right)}^{2}\left(1-\cos\theta \right)d\theta dE$$

where *θ* is the scattering angle, *E* is the carrier energy, *A_j_* is a coefficient related to the scattering mechanism, *q* = 2$\sqrt{2m^{*}\hbar^{-2}E}\sin\left(\theta /2\right)$ is the variation in wave vector during the scattering, *T* is the temperature, and *M_j_* is a matrix element that depends on the types of scattering mechanisms. *S*(*q*,*T,E*) is the screening function describing the 2D screening effect

(12) $$S(q,T,E)=1+\frac{e^{2}F(q)\Pi (q,T,E)}{2\varepsilon q}$$

Here, the form factor *F*(*q*) is expressed as

(13) $$F(q)=\int_{0}^{\infty }\int_{0}^{\infty }\psi^{2}(z)\psi^{2}(z^{\prime })\exp(-q\left|z-z^{\prime }\right|)dzdz^{\prime }$$

Here, *ψ*(*z*) = (*b*^3^*z*^2^/2)^1/2^exp(−*bz*/2) is the Fang–Howard variational wave function and *b* = (12m*e^2^*n*/*ε*ℏ^2^)^1/3^ is a variational parameter, where *n* = *N_depl_* + 11/32 × *n*_2*D*_, and *N_depl_* and *n*_2*D*_ are the depleted electron concentration and the 2DEG density, respectively. The polarizability function Π(*q*,*T*,*E*) is given by

(14) $$\Pi (q,T,E)=\frac{m^{*}}{4\pi \hbar^{2}k_{B}T}\int_{0}^{\infty }\frac{1-u(q-2k_{F}){\left[1-(2k_{F}/q{)}^{2}\right]}^{1/2}}{\cosh^{2}\left[(E_{F}-E)/2k_{B}T\right]}dE$$

where *k_B_* is the Boltzmann constant, *k_F_*=$\sqrt{2m^{*}E_{F}/\hbar^{2}}$ is the Fermi wave vector, *E_F_* = ℏ^2^/(2m*) (3π^2^*N*)^2/3^ is the Fermi potential, and *N* is carrier concentration. *u*(*q* − 2*k_F_*) is a step function.

Equations (11)–(14) are expressions applicable for all types of scattering mechanisms. For individual scattering mechanisms, the matrix element *M_j_* and coefficient *A_j_* are different. If there are multiple scattering mechanisms in a system, the total average freedom time is calculated by the harmonic and Fermi statistical mean method, which is given by

(15) $$\tau_{tot}={\left(\underset{j}{\Sigma }\tau_{j}^{-1}\right)}^{-1}$$

(16) $${\bar{\tau }}_{tot}=\int \tau_{tot}E\frac{\partial f_{0}(E)}{\partial E}dE/\int E\frac{\partial f_{0}(E)}{\partial E}dE$$

where *f*_0_(*E*) is the Fermi–Dirac distribution function.

#### 2.2.2. Phonon Scattering

Phonon scattering originates from the vibration of atoms in the lattice, which is a scattering mechanism that exists in any type of material system. The phonon scattering contains acoustic phonon scattering and optical phonon scattering. The acoustic phonon scattering consists of deformation potential scattering and piezoelectric scattering. The deformation potential scattering rate 1/*τ_dp_* is expressed as [19,20]

(17) $$\frac{1}{\tau_{dp}(\theta ,E)}=\frac{3bE_{dp}^{2}m^{*}k_{B}T}{32\pi \hbar^{3}C_{L}}\iint_{\theta ,E}\frac{\left(1-\cos\theta \right)}{S^{2}(q,T)}dEd\theta$$

where *E_dp_* is the deformation potential and *C_L_* is the longitudinal elastic constant. The scattering rate of the piezoelectric scattering 1/*τ_pe_* is

(18) $$\frac{1}{\tau_{pe}(\theta ,E)}=\frac{{\left(eh_{14}\right)}^{2}m^{*}k_{B}T}{4\pi \hbar^{3}}\iint_{\theta ,E}\frac{\left(1-\cos\theta \right)}{qS^{2}(q,T)}\left[\frac{9}{32C_{L}}f_{L}(y)+\frac{13}{32C_{T}}f_{L}(y)\right]dEd\theta$$

where *h*_14_ is the piezoelectric constant and *C_T_* is the transverse elastic constant. The longitudinal phonons factor is *f_L_*(*y*) = (1 + 6*y* + 12*y*^2^ + 2*y*^3^)/(1 + *y*)^6^, and the transverse phonon factor is *f_T_*(*y*)= (13 + 78*y* + 72*y*^2^ + 82*y*^3^ + 36*y*^4^ + 6*y*^5^)/[13(1 + *y*)^6^], where *y* = *q*/*b*.

In a GaN/AlGaN system, covalent bonds exist alone with ionic bond components, so the optical phonon scattering plays a considerable role. The expression of the polar optical phonon scattering rate 1/*τ_op_* is given by [19,20]

(19) $$\begin{array}{l} \frac{1}{\tau_{op}(\theta ,E)}=\frac{e^{2}m^{*}\hbar \omega_{LO}(\varepsilon_{\infty }^{-1}-\varepsilon^{-1})}{8\pi^{2}\hbar^{3}\left[1-f(E)\right]}\times \\ \iint_{\theta ,E}(1-\cos\theta )\left\{\begin{array}{l} \left[1-f(E)\right]N_{q}I(q_{+})+\left[1-f(E-\hbar \omega_{LO})\right]\\ \times u(E-\hbar \omega_{LO})(N_{q}+1)I(q_{-}) \end{array}\right\}dEd\theta \end{array}$$

Here, ℏ*ω*_LO_ is the longitudinal optical phonon energy, *ε*_∞_ is the optical dielectric constant, and *q*_+_ and *q*_−_ are the wave vectors of the absorption and emission for the phonon, respectively. *N_q_* and *f*(*E*) are the phonon occupation number and Fermi–Dirac distribution function, respectively, given by

(20) $$N_{q}=\frac{1}{\exp(\hbar \omega_{LO}/k_{B}T)-1}$$

(21) $$f(E)=\frac{1}{\exp\left[(E-E_{F})/k_{B}T\right]+1}$$

The quantity *I*(*q*_±_) and the Fang–Howard wave function *I*(*q_z_*)^2^ are defined as

(22) $$I(q_{\pm })=\int (q_{\pm }^{2}+q_{z}^{2}{)}^{-1}{\left|I(q_{z})\right|}^{2}dq_{z}$$

(23) $$I{\left(q_{z}\right)}^{2}=b^{6}/{\left(b^{2}+q_{z}^{2}\right)}^{3}$$

#### 2.2.3. Dislocation Scattering

**Figure 2:**
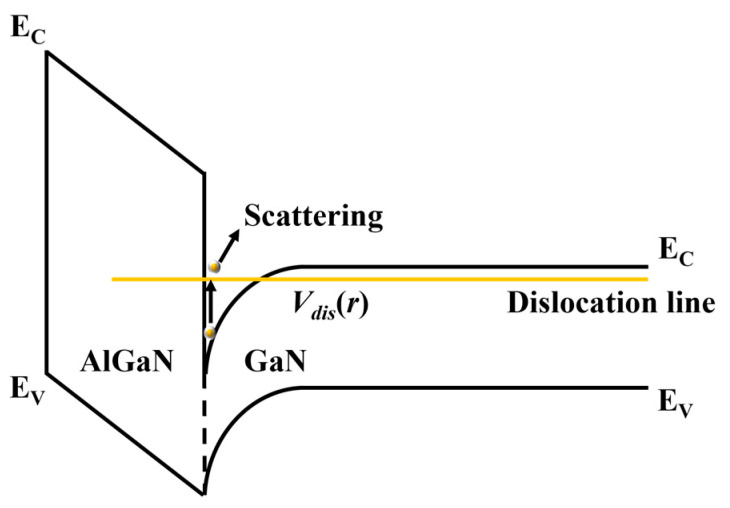
Schematic diagram of dislocation scattering mechanism. Figure depicts energy band of GaN/AlGaN heterostructure when a threading dislocation penetrates through GaN/AlGaN heterojunction. Carriers transport along channel and scatter due to scattering potential *V_dis_*(***r***) generated by dislocation line.

In typical GaN HMET devices, because a GaN/AlGaN heterojunction can generate numbers of dislocations and even penetrate through a GaN/AlGaN heterostructure, the dislocation scattering is a considerable scattering mechanism. Figure 2 shows if a threading dislocation captures electrons, an electron line will be formed around this threading dislocation and generate additional scattering potential *V_dis_*(***r***). 

The analytical formula of dislocation scattering is given by [20]

(24) $$\frac{1}{\tau_{dis}(\theta ,E)}=\left(\frac{m^{*}e^{4}N_{dis}f^{2}}{8\pi \varepsilon^{2}c^{2}\hbar^{3}}\right)\iint_{\theta ,E}{\left\{\int_{z}\psi^{*}(z)\left[\int_{z_{i}}e^{-q\left|z_{i}-z\right|}dz_{i}\right]\psi (z)dz\right\}}^{2}\frac{\left(1-\cos\theta \right)}{{\left[qS(q,T)\right]}^{2}}dEd\theta$$

Here, *N_dis_* is the dislocation density, *f* is the filling factor (usually taken as 1 [21]), and *c* is the lattice constant.

#### 2.2.4. Interface Roughness Scattering

**Figure 3:**
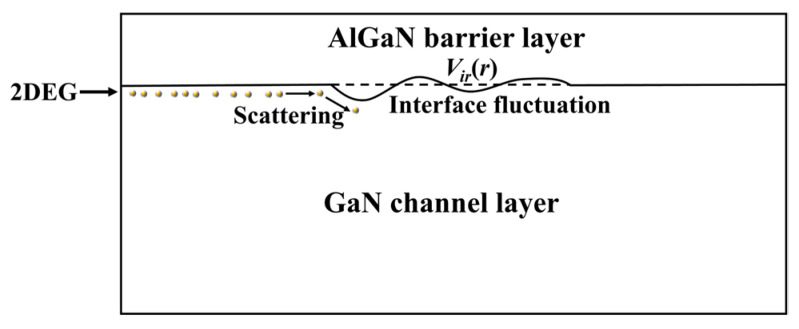
Schematic diagram of interface roughness scattering mechanism. Fluctuation at GaN/AlGaN heterojunction generates a local potential *V_ir_*(***r***) and leads to carrier scattering at GaN/AlGaN interface.

The interface roughness of the GaN/AlGaN heterojunction mainly comes from non-uniformity during film growth. This nonuniformity will lead to slight height fluctuations at the GaN/AlGaN heterojunction (Figure 3) and generate the local potential *V_ir_*(***r***), which will affect the transmission of carriers along the channel and cause interface roughness scattering. 

It is difficult to directly measure or observe roughness at the GaN/AlGaN interface. However, because the AlGaN surface roughness can also reflect the interface roughness [20], we treated the root mean square (RMS) value as the interface roughness in our calculation. Generally, the height fluctuations on the crystal surface result from grain structures [22]. We assumed that the relationship of the height and the lateral extension on the grain is Gaussian distribution, and the correlation function is expressed as [20,23,24,25]

(25) $$\langle \Delta (r)\Delta (r^{\prime })\rangle =\Delta^{2}\exp\left[-\frac{{\left(r-r^{\prime }\right)}^{2}}{\Lambda^{2}}\right]$$

Here, Δ is the RMS measured by atomic force microscopy (AFM), *r* and *r*′ are the position coordinates of two adjacent points, and Λ is a characteristic length describing the lateral extension of the height correlation. The scattering rate of the interface roughness is given by

(26) $$\frac{1}{\tau_{ir}(\theta ,E)}=\frac{m^{*}{\left(\Delta \Lambda \right)}^{2}}{2\hbar^{3}}{\iint_{\theta ,E}\exp\left(-\frac{q^{2}\Lambda^{2}}{4}\right)\left[\frac{(e^{2}/\varepsilon )(n_{s}/2+N_{depl})}{S(q,T)}\right]}^{2}(1-\cos\theta )d\theta dE$$

The total average scattering time is calculated as

(27) $$\frac{1}{\tau_{tot}}=\frac{1}{\tau_{dp}}+\frac{1}{\tau_{pe}}+\frac{1}{\tau_{op}}+\frac{1}{\tau_{dis}}+\frac{1}{\tau_{ir}}$$

(28) $${\bar{\tau }}_{tot}=\int \tau_{tot}E\frac{\partial f_{0}(E)}{\partial E}dE/\int E\frac{\partial f_{0}(E)}{\partial E}dE$$

Finally, by introducing Equation (28) into Equations (6)–(10), the terahertz response voltage model considering multiple scattering mechanisms can be obtained. Table 1 shows the main parameters for the model calculation.

## 3. Results and Discussion

### 3.1. Model Solution

Here, we numerically solve the response voltage model and investigate the influence of multiple scattering mechanisms on the GaN/AlGaN HEMT THz response model using MATLAB software. From Equations (6)–(10), we found that when the overdrive voltage *U*_0_ is fixed, the material-related parameters can only affect the *f*(*ω*) function. In this section, we mainly study the influence of the scattering mechanism on *f*(*ω*) function. For the five previously discussed scattering mechanisms, the deformation potential scattering, piezoelectric scattering, and polar optical phonon scattering result from the lattice vibration and polarization effects in the GaN/AlGaN heterojunction, which are distinctly influenced by temperature. However, the dislocation density scattering and interface roughness scattering are nearly independent of temperature. Therefore, we firstly investigated the influence of temperature on the intrinsic scattering in the GaN/AlGaN HEMT THz detector. When calculating the scattering rate 1/*τ_j_* and total average freedom time $\bar{\tau }$*_tot_*, the scattering angle *θ* and carrier energy *E* need to be integrated. Theoretically, carriers can possibly scatter to any direction after elastically colliding and distributing at any energy state, and the integration boundaries should be 0 to 2π for *θ* and 0 to ∞ for *E*. However, when *θ* = 0 or 2π, Equation (11) will have infinite value, so the integral range of *θ* is from π/90 to 179π/90, which is within the tolerable solution range. When *E* is larger than 8 × 10^−19^ J (5 eV), the solution will not change with the increase in *E*, so the region of *E* is taken as 0 to 8 × 10^−19^ J. The dislocations, interface roughness, and lateral scale are taken as the typical value of *N_dis_* = 10^10^/cm^2^, Δ = 1 nm, and Λ = 10 nm, respectively.

**Figure 4:**
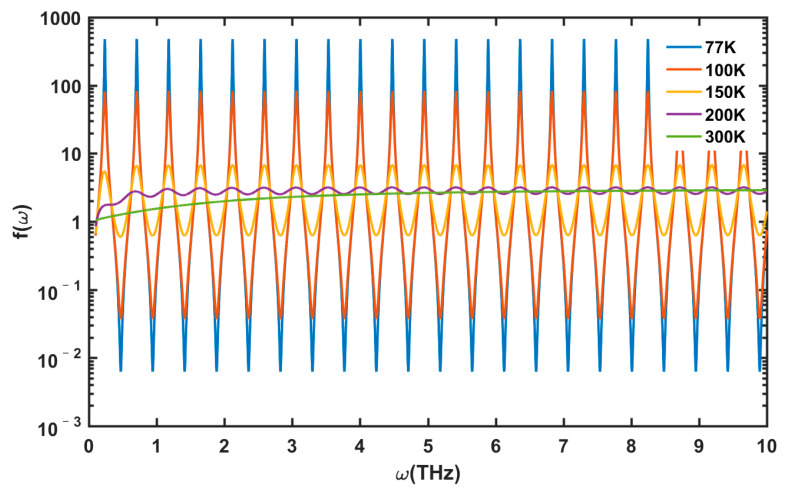
Variations in *f*(*ω*) with temperature in frequency range of 0.1–10 THz. Other parameters are taken as *n_s_* = 1 × 10^13^/cm^2^ *N_dis_* = 10^10^/cm^2^, Δ = 1 nm, and Λ = 10 nm.

Figure 4 shows the *f*(*ω*) function with the change in the incident THz frequency from 0.1 to 10 THz at several typical temperatures. At a low-temperature range (*T* < 150 K), an obvious resonance state can be observed. The resonant fundamental frequency is *ω_F_* =π*s*/(2*L*), and *s* is taken as a typical value of 3 × 10^7^ cm/s. From a relatively high temperature to room temperature (150 K < *T* < 300 K), the resonant phenomenon gradually weakens until it totally disappears. In a low-temperature region, atoms are at a low-energy state, the lattice vibrational frequency is relatively low, and the acoustic phonon scattering (deformation potential scattering and piezoelectric scattering) plays a dominant role without optical phonon scattering. At this condition, the plasmon loss in the channel is small, so the plasmon resonance is considerable. At high temperature, the lattice vibrational frequency is higher, and the optical phonon scattering is not negligible and even increases sharply when the temperature is close to room temperature. Therefore, the total scattering and plasmon-loss rates increase, and the detection mode transforms from a resonant to non-resonant state.

**Figure 5:**
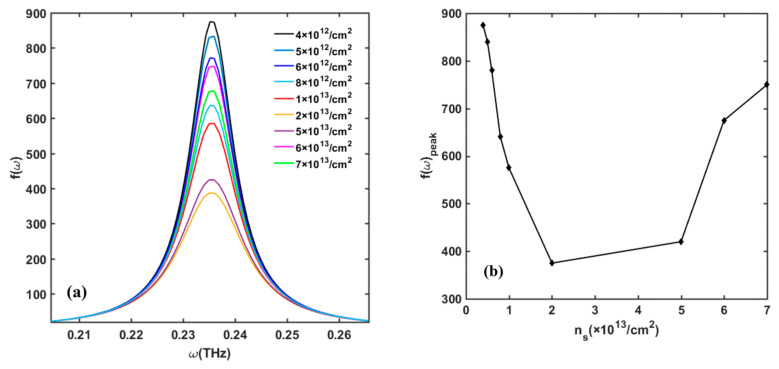
(**a**) Variation in *f*(*ω*) with 2DEG density *n_s_* in frequency range of 0.1–10 THz at temperature of 77 K. (**b**) Peak value of *f*(*ω*) with change in *n_s_*. Other parameters are taken as *N_dis_* = 10^10^/cm^2^, Δ = 1 nm, and Λ = 10 nm.

**Figure 6:**
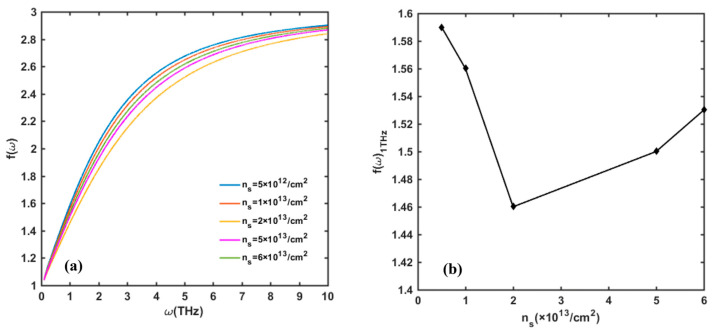
(**a**) Variation in *f*(*ω*) with 2DEG density *n_s_* in frequency range of 0.1–10 THz at temperature of 300 K. (**b**) Value of *f*(*ω*) at 1 THz incident frequency with change in *n_s_*. Other parameters are taken as *N_dis_* = 10^10^/cm^2^, Δ = 1 nm, and Λ = 10 nm.

Figure 5 displays the value of the *f*(*ω*) function versus 2DEG surface density *n_s_* when the detector operates in resonant detection mode (at liquid-nitrogen temperature *T* = 77 K). The peak value of the *f*(*ω*) function at the fundamental frequency and its harmonics first decreases and then increases with the 2DEG surface density. At low temperature, deformation scattering, especially piezoelectric scattering, dominates. With the increase in *n_s_*, the piezoelectric scattering is enhanced and the loss of plasmon increases. However, when the *n_s_* reaches 5 × 10^13^/cm^2^, the *f*(*ω*)_peak_ starts to increase. This is because the number of plasmons will increase with *n_s_*, which will aggravate the resonance in the channel. Figure 6 depicts the change in *f*(*ω*) function with *n_s_* when the detector operates as a non-resonant mode. At room temperature, optical phonon scattering is the dominant mechanism. We observe that the *f*(*ω*) function shows similar phenomena with the resonant mode. At a specific frequency, such as 1 THz, *f*(*ω*) first decreases and then increases with the increase in *n_s_*. Notably, *n_s_* is related to various factors, such as Al component, barrier-layer thickness, gate-to-source voltage, etc. To simplify the model, we give the value of *n_s_* directly instead of specifying its source. In practical experiments, *n_s_* can hardly achieve above 5 × 10^13^/cm^2^, so the gate voltage is usually set close to the threshold voltage *V_th_* to reduce *n_s_* and maximize the detector’s response voltage.

**Figure 7:**
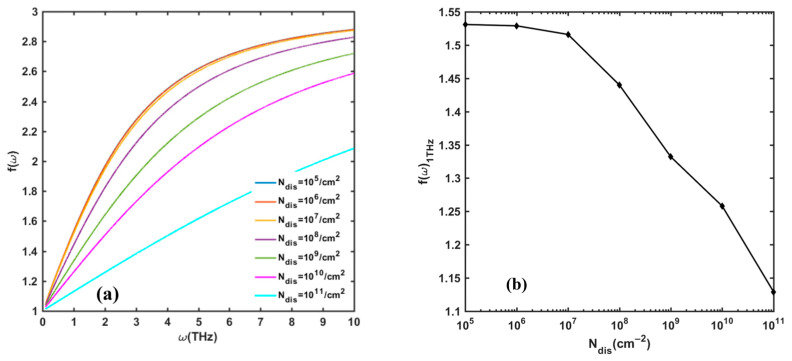
(**a**) The variation in *f*(*ω*) with dislocation density *N_dis_* in frequency range of 0.1–10 THz. (**b**) Value of *f*(*ω*) at 1 THz incident frequency with change in *N_dis_*. Other parameters are taken as *n_s_* = 10^13^/cm^2^, Δ = 1 nm, and Λ = 10 nm.

Figure 7 describes the effect of dislocation density on *f*(*ω*) at room temperature. Apparently, the smaller the dislocation density is, the better the detector’s response performance. Generally, the threading dislocation in a GaN/AlGaN film system includes screw, edge, and mixed dislocations. The literature [22,26,27] and our previous experimental results [28] prove that different types of dislocations can induce different scattering potentials, which also have different effects on carrier transport capacity. In this model, we consider the total number of dislocations to calculate the response voltage. Our results can evaluate the overall effect of dislocation density on response performance. If the dislocation density is smaller than 10^7^/cm^2^, the dislocation scattering mechanism can be ignored; however, if the dislocation density is above 10^10^/cm^2^, the influence of the dislocation scattering mechanism will be considerable.

**Figure 8:**
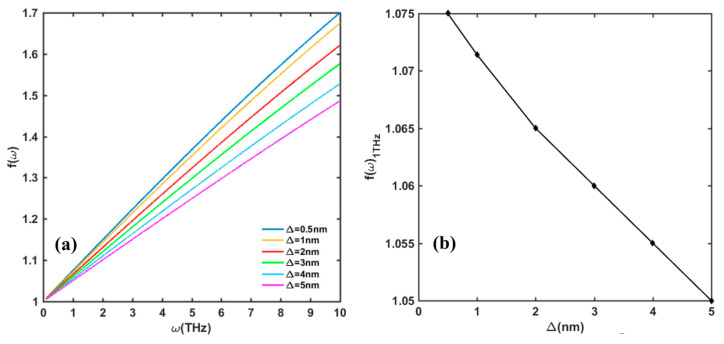
(**a**) Change in *f*(*ω*) with interface roughness Δ in frequency range of 0.1–10 THz. (**b**) Value of *f*(*ω*) at 1 THz incident frequency with change in Δ. Other parameters are taken as *n_s_* = 10^13^/cm^2^, *N_dis_* = 10^10^/cm^2^, and Λ = 10 nm.

**Figure 9:**
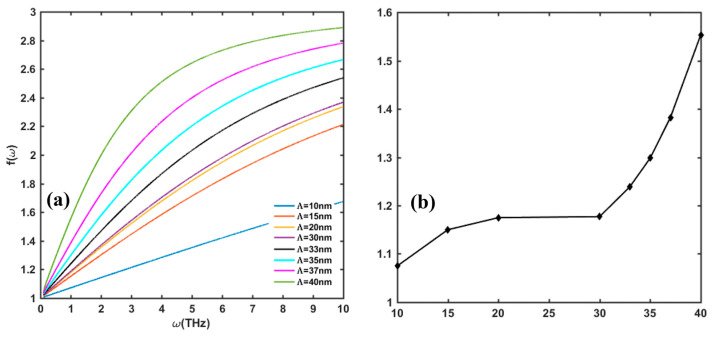
(**a**) Variation in *f*(*ω*) with lateral correlation length Λ in frequency range of 0.1–10 THz. (**b**) Value of *f*(*ω*) at 1 THz incident frequency with change in Λ. Other parameters are taken as *n_s_* = 10^13^/cm^2^, *N_dis_* = 10^10^/cm^2^, and Δ = 1 nm.

The effect of interface scattering on *f*(*ω*) function is demonstrated in Figure 8 and Figure 9. As discussed in Section 2, there are two parameters that describe the interface roughness, the roughness height ∆, and the lateral correlation length Λ. Figure 8 shows that at a certain Λ, the value of *f*(*ω*) gently decreases with the increase in ∆. Figure 9 shows that *f*(*ω*) dramatically increases with the increase in Λ, especially when Λ is above 30 nm. When Λ is above 40 nm, the influence of the interface scattering mechanism is negligible. In reality, the measured RMS value is related to the scanning area. Generally, the smaller the scanning area, the smaller RMS will be, so the measured RMS can only reflect a morphology of the local area instead of the whole surface morphology. However, Figure 8 shows that when ∆ changes from 0.5 nm to 5 nm, the value of *f*(*ω*) only varies in a small range. The interface roughness scattering is mainly related to the crystal’s grain size, which depends on the growing process of the GaN/AlGaN epitaxial layer.

### 3.2. Model Validation

**Figure 10:**
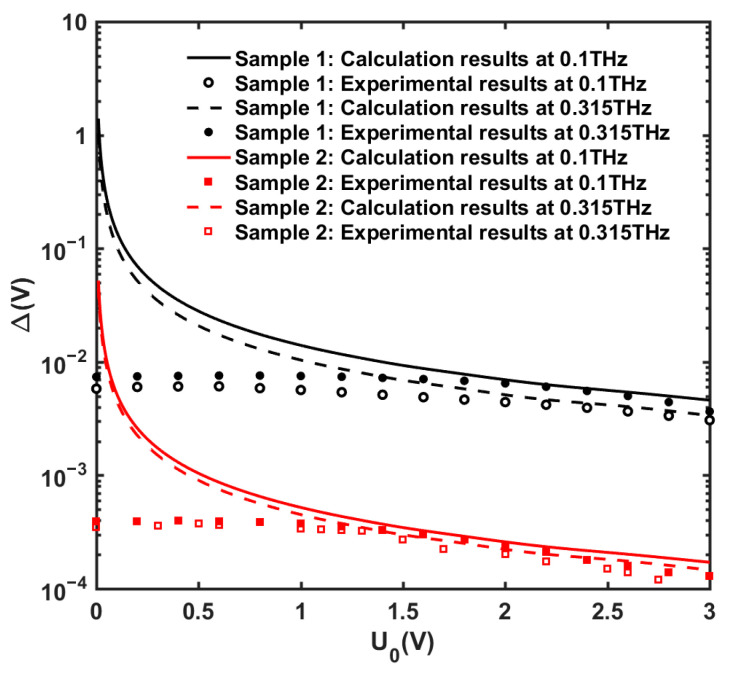
Comparison between experimental results of prepared samples and theoretical calculation results at incident THz radiation frequency of 0.1 THz and 0.315 THz.

Next, we use the experimental results [14,28] of our two prepared GaN/AlGaN HEMT terahertz detector samples to verify the accuracy of our proposed model. It should be clarified that our model can only predict the response voltage instead of the responsivity and noise equivalent power (NEP) of the detector. This is because the power and effective area of the incident THz radiation have to be calibrated in the experiment, and the minimum background noise is estimated by the standard quantum limit (SQL), which is out of the scope of this work. Here, we compared the measured response voltage of the samples with the solution of the model. The response voltage with the change in gate–source voltage *V_gs_* for two samples was measured at the incident THz frequencies of 0.1 THz and 0.315 THz. To compare the experimental and calculation results of our proposed model under the same conditions, the variable *s* related to *V_gs_* is replaced by *s* = (*eU*_0_/*m*)^1/2^ in Equations (6)–(10). The samples’ material parameters are listed in Table 2. Figure 10 shows a comparison between the experimental and calculated results of the response voltage for the two samples. When the overdrive voltage *U*_0_ is close to zero, the calculated response voltage is extremely high since the model has a singular point with infinite value at *U*_0_ = 0 (in Equation (6)). However, due to the thermal motion of the carriers, *U*_0_ will never be zero in reality. When *U*_0_ is larger, the calculated results are basically consistent with the experimental results. Figure 10 shows that the response voltage of Sample 2 is larger than 1, regardless of the incident frequency at 0.1 THz or 0.315 THz. Table 2 shows that the 2DEG densities of Sample 1 and 2 are almost equal, and the dislocation density of 1 is about twice that of 2, while the RMS and lateral correlation length are both smaller than that of 1. As previously analyzed in Section 3.1, the smaller the dislocation density and the larger the lateral correlation length are, the better the response voltage will be, while the effect of RMS on the response voltage is weak. Consequently, Sample 2 shows better detection performance than 1. The overall results demonstrate that our proposed model obtains similar trends with the experimental results. The small discrepancy between the calculated and experimental results is due to the coupling efficiency of the THz antenna and the difference of dislocation types. The assumption for the proposed THz response voltage model is that the incident THz wave is completely absorbed and coupled into the channel of the GaN HEMT THz detector. The incident THz radiation is absorbed by a THz antenna and then coupled into the channel. In this process, the return loss of the antenna makes a portion of THz radiation power not fully coupled. For dislocation scattering, we used the total dislocation density to calculate the scattering rate and simplify the model. In reality, the location distribution of the dislocations and their different types do not contribute to dislocation scattering. These two factors overestimate the calculated THz response voltage, so the experimental results are a little lower than the calculated results. 

To further investigate the accuracy of our proposed model, we compared the model error with the current FET THz detection models. The model error is defined as the percentage error between the calculated and experimental results. The main parameters used in the calculation are shown in Table 3. Because there are hardly any FET THz detection models that contain the appropriate material parameters, such as dislocation density or surface roughness, the material parameters are represented by *τ*. To compare model errors under the same conditions, we substitute the parameters of other models into our model for error calculation. Compared with the reported models, our proposed model has a smaller error rate. At room temperature and low-incident THz frequency, *τ* decreases with the increase in gate length *L*, and our proposed model has smaller *τ*. This is because the carrier scattering mechanism is considered in the model, which is closer to the experimental results. In the low-temperature range, due to the dominant deformation potential scattering, the dislocation scattering and interface roughness scattering have little influence, so the error rate of the model is relatively small. In general, our proposed model has high accuracy and can be used to predict the performance of GaN HEMT THz detectors.

## 4. Conclusions

This paper proposed a THz response voltage model for a GaN HEMT THz detector affected by the influence of carrier scattering mechanisms. The effect of material parameters on the detection model, including temperature, 2DEG density, dislocation density, and interface roughness, were numerically investigated, and the accuracy of the proposed model was verified by the experimental results of our prepared GaN HEMT THz detector samples. Our comparison of the experimental and calculation results indicated that our proposed model obtained similar trends to the experimental results, which were basically consistent with each other. In addition, our proposed model has a smaller error rate than the current reported models. Generally, our proposed model is accurate enough to evaluate the effect of carrier scattering on the response voltage for GaN HEMT THz detectors.

## Figures and Tables

**Table 1:** Parameters used in calculation.

Parameters	Value
Gate length	*L* = 2 μm
Effective electron mass	m* = 0.22*m*_0_
Electron charge	*e =* 1.6 × 10^−19^ C
Boltzmann constant	*k_B_* = 1.38 × 10^−23^ J/K
Dielectric constant (low frequency)	*ε* = 10.4*ε*_0_
Deformation potential	*E_dp_ =* 8.5 eV
Piezoelectric constant	*h_14_* = 4.3 × 10^9^ V/m
Longitudinal elastic constant	*C_L_ =* 2.65 × 10^11^ N/m^2^
Transverse elastic constant	*C_T_* = 4.42 × 10^10^ N/m^2^
LO-phonon energy	ℏ*ω*_LO_ = 91.2 meV
Dielectric constant (high frequency)	*ε*_∞_ = 5.47*ε*_0_
Filling factor	*f* = 1
Lattice parameter	*c* = 5.185 Å

**Table 2:** Main material parameters of prepared samples.

	2DEG Density (cm^−2^)	Dislocation Density (cm^−2^)	RMS (nm)	Lateral Correlation Length (nm)
Sample 1	1.019 × 10^13^	4 × 10^9^	0.402	28.72
Sample 2	9.247 × 10^12^	1.8 × 10^9^	0.538	38.67

**Table 3:** Main material parameters and model error.

Model Type	*L* (μm)	*ω* (THz)	*τ* (ps)	*T* (K)	Ref.	Error for Other’s Model	Error for This Model
Model considering leakage current	0.1	0.674	0.0741	300	[15]	9.7%	7.3%
Classic drift-diffusion model	0.3	0.7	0.0377	300	[29]	6.4%	5.1%
Classic drift-diffusion model	2	0.2	~0.02	300	[30]	8.3%	7%
Model considering loading effect	0.25	0.23	~0.1 ~0.3	250 150	[16]	5% 5.5%	4.1% 4.4%
Model in sub-threshold region	5	0.2	~0.15	200	[31]	5.8%	4.5%
Model considering scattering mechanisms	2	0.1 0.315	0.0134	300	This work		4.8% 4.7%

## Data Availability

All data are contained within this article.
